# Integrative analysis of ceRNA network reveals functional lncRNAs associated with independent recurrent prognosis in colon adenocarcinoma

**DOI:** 10.1186/s12935-021-02069-6

**Published:** 2021-07-05

**Authors:** Yinling Mao, Jiachen Lv, Li Jiang, Yihui Wang

**Affiliations:** 1 Department of Abdominal Radiotherapy, Harbin Medical University Cancer Hospital, Harbin, 150001 Heilongjiang Province China; 2 Department of Colorectal Surgery, Harbin Medical University Cancer Hospital, NO. 150 Hapin Road, Harbin, 150001 Heilongjiang Province China; 3 Department of Hemolymph, Harbin Medical University Cancer Hospital, Harbin, 150001 Heilongjiang Province China

**Keywords:** Colon adenocarcinoma, lncRNA signature, Risk score, Nomogram survival model, Competitive endogenous RNA, Recurrence prognosis

## Abstract

**Background:**

Long non-coding RNAs (lncRNAs), acting as competing endogenous RNA (ceRNA) have been reported to regulate the expression of targeted genes by sponging miRNA in colon adenocarcinoma (COAD).

**Methods:**

However, their potential implications for recurrence free survival prognosis and functional roles remains largely unclear in COAD. In this study, we downloaded the TCGA dataset (training dataset) and GSE39582 (validation dataset) of COAD patients with prognostic information.

**Results:**

A total of 411 differentially expressed genes (DElncRNAs: 12 downregulated and 43 upregulated), 18 DE miRNAs (9 downregulated and 9 upregulated) and 338 DEmRNAs (113 downregulated and 225 upregulated) were identified in recurrence samples compared with non-recurrence samples with the thresholds of FDR < 0.05 and |log_2_FC|> 0.263. Based on six signature lncRNAs (LINC00899, LINC01503, PRKAG2-AS1, RAD21-AS1, SRRM2-AS1 and USP30-AS1), the risk score (RS) system was constructed. Two prognostic clinical features, including pathologic stage and RS model status were screened for building the nomogram survival model. Moreover, a recurrent-specific ceRNA network was successfully constructed with 2 signature lncRNAs, 4 miRNAs and 113 mRNAs. Furthermore, we further manifested that SRRM2-AS1 predicted a poor prognosis in COAD patients. Furthermore, knockdown of SRRM2-AS1 significantly suppressed cell proliferation, migration, invasion and EMT markers in HT-29 and SW1116 cells.

**Conclusion:**

These identified novel lncRNA signature and ceRNA network associated with recurrence prognosis might provide promising therapeutic targets for COAD patients.

**Supplementary Information:**

The online version contains supplementary material available at 10.1186/s12935-021-02069-6.

## Background

Colon cancer is currently the most common type of gastrointestinal malignancy with increasing 4.2% incidence globally every year [1, 2], which is histologically divided into colon adenocarcinoma (COAD), undifferentiated carcinoma, and mucinous adenocarcinoma [3]. As the most common subtype of colon cancer, COAD is defined as type of malignant epithelial tumor from normal mucosa to adenoma and finally to carcinoma [4, 5]. It has been reported that the overall five-year survival rate for COAD, particularly for patients at advances stages, is less than 40%, which is largely ascribed to post-operative recurrence and metastasis [6–8]. Therefore, the identification of valuable molecular markers associated with recurrence may guide early prediction and treatment of COAD patients.

Long non-coding RNAs (lncRNAs) is known as a class of RNA molecules with over 200 nucleotides in length and have no evident open reading frames without non-protein coding ability [9]. It has been certified that lncRNAs are dysregulated in the progression of various cancers and play vital roles in gene regulation and carcinogenesis, including proliferation, migration and genomic stability [10, 11]. Accumulating evidence has confirmed the oncogenic or tumor suppressive role of lncRNAs in colon cancer development. For instance, lncRNA CASC15 promotes colon cancer cell proliferation and metastasis by regulating the miR‑4310/LGR5/Wnt/β‑catenin signaling pathway [12]. LncRNA B3GALT5-AS1 suppressed colon cancer liver metastasis via its binding on miR-203 promoter and the repression of miR-203 [13]. Recently, several studies based on the development of bioinformatics have developed lncRNA-related signatures for predicting prognosis of colon cancer. As reported by Fu et al. [14], a seven-lncRNA signature associated with prognosis of COAD was identified and validated by different cohorts. Lin et al. [15] developed an immune-related nine-lncRNA signature predictive of overall survival in colon cancer. In addition, Zhou et al. [16] identified ten prognostic autophagy-related lncRNAs, which made up an autophagy-related lncRNA signature as therapeutic targets for the COAD patients. Nevertheless, the research focused on functional lncRNAs associated with independent recurrent prognosis still remain relatively little.

A key regulatory mechanism for lncRNAs is the competitive endogenous RNA (ceRNA) hypothesis which bind to microRNA (miRNAs) through miRNA response elements (MREs), thereby regulating miRNAs‐induced gene silencing [17]. Based on ceRNA theory, Wu et al. [18] revealed lncRNA MALAT1 may serve as a competing endogenous lncRNA (ceRNA) to mediate HMGB1 by sponging miR-129-5p in colon cancer. Liu et al. [19] demonstrated that SNHG17 serves as competing endogenous RNA (ceRNA) for miR-375 to regulate CBX3 expression in COAD. We thus believe lncRNAs functions as ceRNAs deserve further exploration in exploring the molecular mechanism underlying COAD recurrent prognosis.

In present research based on TCGA and GEO database, we analyzed the differentially expressed RNAs (DERs), including DElncRNAs, DEmiRNAs and DEmRNAs between recurrence and non-recurrence COAD samples. By screening independent recurrence prognosis-related lncRNAs, we constructed risk score system and nomogram survival model. Moreover, we established ceRNA regulatory network associated with lncRNA signature and conducted enrichment analysis to elucidate the interactions and valid potential crosstalk between RNAs.

## Methods

### Microarray datasets

Gene expression profiles and corresponding clinical data of COAD patients (Platform: Illumina HiSeq 2000 RNA Sequencing) were downloaded from The Cancer Genome Atlas (TCGA, http://cancergenome.nih.gov/) on April 20, 2019, including 465 tumor tissues and 85 normal tissues. After matching with 461 miRNA expression profiles downloaded at the same time (Illumina Hiseq 2000 RNA Sequencing), a total of 363 COAD samples with recurrence information (78 recurrence and 285 non-recurrence) constituted the training dataset. For validation propose, we searched microarray data from Gene Expression Omnibus (https://www.ncbi.nlm.nih.gov/geo/) database with the following criteria: 1) Tumor solid tissue samples from patients with COAD; 2) The sample size more than 200; 3) The COAD tumor samples had clinical information on the prognosis of recurrence. Finally, microarray data of GSE39582 including 536 COAD patients with recurrence information were obtained under the platform GPL570 Affymetrix Human Genome U133 Plus 2.0 Array.

### Analysis of differential expressed RNAs (DERs)

According to downloaded RefSeq ID information from the training set and the validation set, the lncRNAs and mRNAs of the two sets were annotated based on HUGO Gene Nomenclature Committee (HGNC) [20] (http://www.genenames.org/), the database which records information of 4120 lncRNAs and 19,198 protein-coding genes. Then, differentially expressed DERs, including DElncRNAs, DEmiRNAs and DEmRNAs were screened between recurrence and non-recurrence specimens using the limma package (software version 3.34.7) of R [21]. False discovery rate (FDR) < 0.05 and |log_2_ fold change (FC)|> 0.263 (FC > 1.2) were set as the cutoff for significance. Two-way hierarchical clustering analysis based on Centered Pearson Correlation Algorithm [22] was carried out for these identified DERs by pheatmap Version 1.0.8 in R3.4.1 language [23].

### Construction and validation of prognostic predictive model

Using survival package Version 2.41–1 in R3.4.1 language [24], these DElncRNAs were subjected to univariable and multivariable Cox regression proportional hazards regression analysis to select the independent risk DElncRNAs and obtain corresponding coefficients in training dataset with log-rank *p* value < 0.05. Next, least absolute shrinkage and selection operator (LASSO) Cox regression model [25] in penalized package Version 0.9.50 of R3.4.1 language [26] was used to screen independent risk signature lncRNAs (The optimized parameter "lambda" in the screening model is obtained by the cross-validation likelihood (CVL) cycle calculation of 1000 times). By linearly combining the expression value of selected signature lncRNAs weighted by their coefficients, a risk-score (RS) formula was constructed as following: RS = ∑β_lncRNA_ × Exp_lncRNA_, where β_lncRNA_ indicates the coefficient and Exp_lncRNA_ indicates the expression level of signature lncRNA. The RS of every patient from the TCGA and GEO cohorts were calculated based on the signature. The subjects in each dataset were classified into a high-risk group and low-risk group with the median score as cut-off value. We used the Kaplan–Meier method with log-rank test in R3.4.1 survival package Version 2.41–1 [24] to perform recurrence free survival (RFS) analysis for each set. The receiver operating characteristic (ROC) curve and the area under the curve (AUC) were drawn using R package “survival ROC” and utilized to validate the prediction model.

### Construction of nomogram survival model for independent prognostic factor

We first screened the independent prognostic factors in the training dataset with the significance threshold of log-rank p < 0.05 by performing the univariate and multivariate regression analysis in the survival package of R3.4.1 (version 2.41–1). Afterwards, the identified independent prognostic factors were combined with the predicted risk information in the prediction prognosis model to construct a nomogram 3-year or 5-year survival rate model using R3.4.1 rms package Version 5.1–2 (https://cran.r-project.org/web/packages/rms/index.html) [27, 28]. Finally, we compared the actual and predicted probabilities of 3-year RFS and 5-year RFS using calibration plots.

### Construction of ceRNA regulatory network

We first used DIANA-LncBasev2 database (http://carolina.imis.athena-innovation.gr/diana_tools/web/index.php?r=lncbasev2%2Findex-experimental) [29] to predict the interactions between six-lncRNA signature and DEmiRNAs. Secondly, starBase version 2.0 database (http://starbase.sysu.edu.cn/) [30] was searched to predict the corresponding target mRNAs of selected DEmiRNAs. Meanwhile, the regulatory relationships included in at least one of five databases (TargetScan, PicTar, RNA22, Pita and MIRANDA) were selected as target mRNAs. Then, the DEmRNAs were corresponded into the target mRNAs. Only the regulatory pairs of DElncRNAs and DEmiRNAs, DEmiRNAs and DEmRNAs had opposite expressions and therefore included in the present study. Finally, the selected interaction of DEmiRNAs and DEmRNAs and of DElncRNAs and DEmiRNAs were integrated to construct the ceRNA regulatory network using Cytoscape 3.6.1 visualization software (https://cytoscape.org/) [31].

### Function enrichment analysis

To better understand the function of DERs in ceRNA network, we performed Kyoto Encyclopedia of Genes and Genomes (KEGG) functional enrichment analyses based on gene set enrichment analysis (GSEA: http://software.broadinstitute.org/gsea/index.jsp) [32]. With the nominal p < 0.05 as the cut-off criteria, we chose signature lncRNAs with significant enrichment and displayed gene sets enrichment plots.

### Clinical tissues

Total 60 paired tumor tissues and adjacent normal tissues from COAD patients were collected from Harbin Medical University Cancer Hospital (Heilongjiang, China) after two pathologists independently diagnosed the pathological features of the tumor tissues. Some basic clinicopathological characteristics of COAD patients, including age, gender and TNM stage, as well as follow-up information were recorded. None of patients received any neoadjuvant chemotherapy or radiotherapy before operation. The study was approved by the Ethics Committee of the Harbin Medical University Cancer Hospital with signed written informed consent by all subjects.

### Cell culture and transfection

Four COAD cell lines (HT-29, DLD-1, SW1116 and RKO) and normal human colon epithelial cell line (FHC) were purchased from American Type Culture Collection (ATCC, Manassas, VA, USA). All cell lines were cultured in DMEM medium (Gibco, Carlsbad, CA) with 10% fetal bovine serum (FBS, Gibco) in a humidified atmosphere of 37 °C containing 5% CO_2_.

The small interference RNAs (siRNA) for SRRM2-AS1 (si-SRRM2-AS1) and negative control (si-NC) were produced by GenePharma Company (Shanghai, China) for the depletion of SRRM2-AS1 in HT-29 and SW1116 cells. Cell transfection was achieved using Lipofectamine 3000 (Invitrogen, Carlsbad, CA). Samples were harvested after 48 h of transfection.

### Quantitative RT-PCR

Total RNA was extracted using TRIzol (Invitrogen) and reverse transcription was performed with PrimeScript RT reagent kit (Takara, Otsu, Japan) or TaqMan miRNA Reverse Transcription Kit (Takara) according to the manufacturer’s instructions. Quantitative RT-PCR was conducted using Power SYBR Green (TaKaRa) on StepOnePlus system (Applied Biosystems) with the following primer sequences: PRKAG2-AS1 forward: 5ʹ‐CCCAACTAGACACCTACATCC‐3ʹ and reverse: 5ʹ‐GCTTGATCTCTACCCTTGCTT‐3ʹ; SRRM2-AS1 forward: 5ʹ-TCCTGCTATCGCTTCCCAGT‐3ʹ and reverse: 5ʹ-GGTTGCGACGTAATAGGAAGGT‐3ʹ; STRADA, forward: 5′‐CGGGTGACACTCGGAGAAAA‐3′, reverse: 5′‐AGTGAGCAGCTCGTAACACC‐3; GAPDH forward: 5ʹ-GGAGCGAGATCCCTCCAAAAT‐3ʹ and reverse: 5ʹ‐GGCTGTTGTCATACTTCTCATGG‐3ʹ; miR‐6514, forward: 5′‐TATGGAGTGGACTTTCAGCTGGC‐3′, reverse: 5′‐CTGGAGTGGAAGAACAGGCA‐3′; miR‐1275, forward: 5′‐TGGGGGAGAGGCTGTC‐3′, reverse: 5′‐GAACATGTCTGCGTATCTC‐3′; U6, forward: 5′‐CTCGCTTCGGCAGCACAT‐3′, reverse: 5′‐TTTGCGTGTCATCCTTGCG‐3′; The relative expression level was calculated with 2^−ΔΔCt^ method with GAPDH or U6 as the internal reference for normalization.

### Cell proliferation assay

Transfected cells at a density of 5,000 cells per well were seeded in a 96‐well plate and 10 μl Cell Counting Kit-8 reagent (Dojindo Molecular Technologies, Inc.) was added to each well. After incubated at different times (0, 24, 48 and 72 h), the absorbance at 450 nm was measured to represent cell proliferation status.

### Cell migration and invasion assays

For cell migration assay, 200 μL serum-free medium of transfected cells (8 × 10^4^) was placed in the upper inserts of transwell chamber (8 μm pore size, Corning, Corning, NY, USA). Meanwhile, 700 μL of medium containing 10% FBS was added into the lower inserts of transwell as a chemical attractant. After 24 h incubation, the migratory cells in lower chamber were fixed in methanol and stained with 0.1% crystal violet, followed by cell counting in five randomly selected fields under the microscope. For cell invasion assay, the procedure of invasion assay was similar with that of migration assay, except for the transwell chamber precoated with Matrigel (BD Biosciences).

### Western blot analysis

Protein from cell lines were extracted using 1 × cell lysis buffer (Promega, Madison, WI, USA). The SDS-PAGE electrophoresis and immunoblotting were performed as previously reported [33] with antibodies specific for E-cadherin, Vimentin, Snail, glyceraldehyde-3-phosphate dehydrogenase (GAPDH; Santa Cruz Biotechnology, Santa Cruz, CA, USA).

### Statistical analysis

The statistical analysis software performed was GraphPad Prism 6.0 (GraphPad Software, Inc., USA). The chi-square test was used to assess the relationship between SRRM2-AS1 expression and clinicopathological features of COAD patients. Overall survival was analyzed by the Kaplan–Meier method and compared by the log-rank test. The significance of various variables for survival data was evaluated by univariate and multivariate Cox regression models. Quantitative data were expressed as mean ± SD and analyzed for significant difference in two groups by Student’s t test. All data with *p* values less than 0.05 were recognized as statistically significant.

## Results

### Identification of DERs

**Fig. 1:**
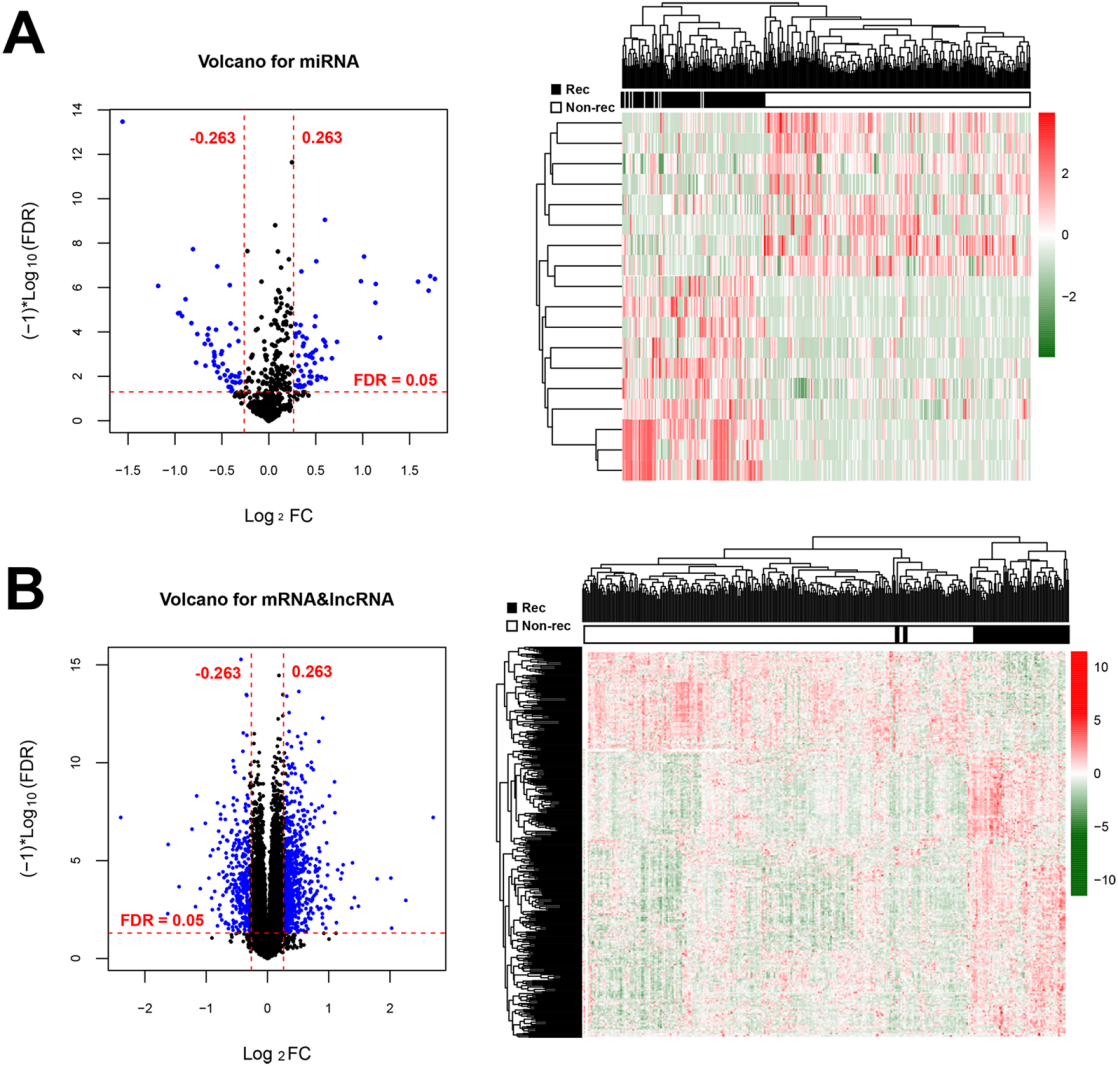
Volcano plot and bidirectional hierarchical clustering heatmap. A Left picture: Volcano plot depicting the DEmiRNAs; Blue dots indicate the DEmiRNAs. The red horizontal dotted line indicates FDR < 0.05 and two red vertical dashed lines indicate |log_2_FC|> 0.263. Right picture: Bidirectional hierarchical clustering heat map based on DEmiRNAs; The white and black samples below represent non-recurrence and recurrence samples, respectively. **B** Left picture: Volcano plot depicting the DEmRNAs and DElncRNAs; Blue dots indicate the DEmRNAs and DElncRNAs. The red horizontal dotted line indicates FDR < 0.05 and two red vertical dashed lines indicate |log_2_FC|> 0.263. Right picture: Bidirectional hierarchical clustering heat map based on DEmRNAs and DElncRNAs; The white and black samples below represent non-recurrence and recurrence samples, respectively

Following data annotation, we obtained 549 miRNAs, 12,008 mRNAs and 827 lncRNAs overlapped by the TCGA set and the validation set. Based on the screening criteria of |log_2_FC|> 0.263 and FDR < 0.05, DERs, including 18 DEmiRNAs (9 up-regulated and 9 down-regulated), 338 DEmRNAs (225 up-regulated and 113 down-regulated) and 55 DElncRNAs (43 up-regulated and 12 down-regulated) were identified in recurrence samples compared with non-recurrence samples (Additional file 1: Table S1). Volcano plot and bidirectional hierarchical clustering heatmap were described to the DEmiRNAs (Fig. 1A), DEmRNAs and DElncRNAs (Fig. 1B), which clearly indicated the samples tend to cluster in two distinct directions.

### Construction and validation of 6 lncRNA-based prognostic signature

**Fig. 2:**
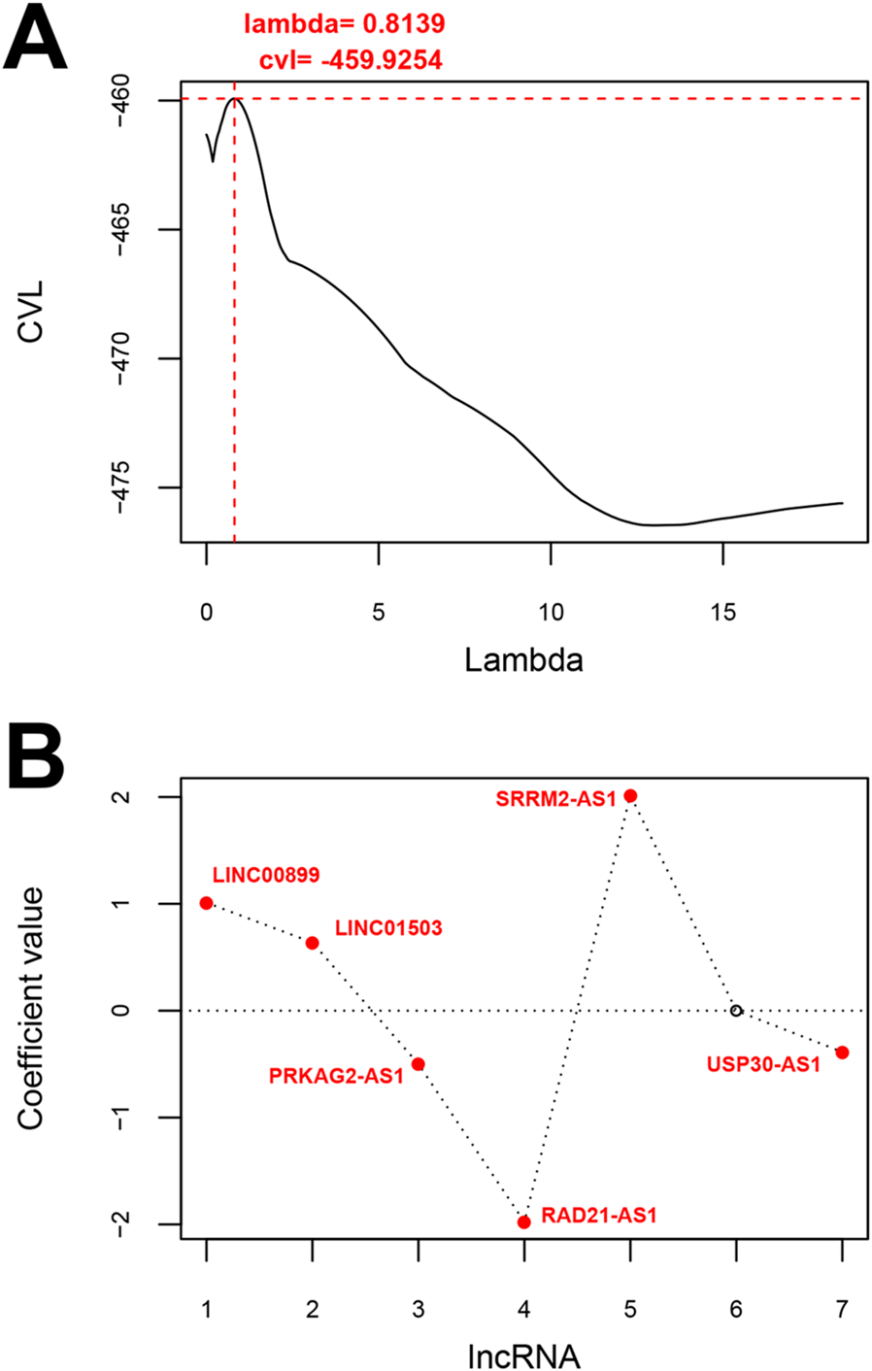
Identification of 6 signature lncRNAs significantly and independently correlated to prognosis. **A** Cross-validation likelihood filtering for lambda parameter curves. The horizontal axis and the vertical axis represent the different values of lambda and CVL, respectively. The red dotted line intersection indicates the value of lambda parameter (0.8139) when CVL takes the maximum value (− 459.9254). **B** Distribution map of gene coefficients related to optimal prognosis screened by COX-PH model based on the L1-penalized regression algorithm

**Fig. 3:**
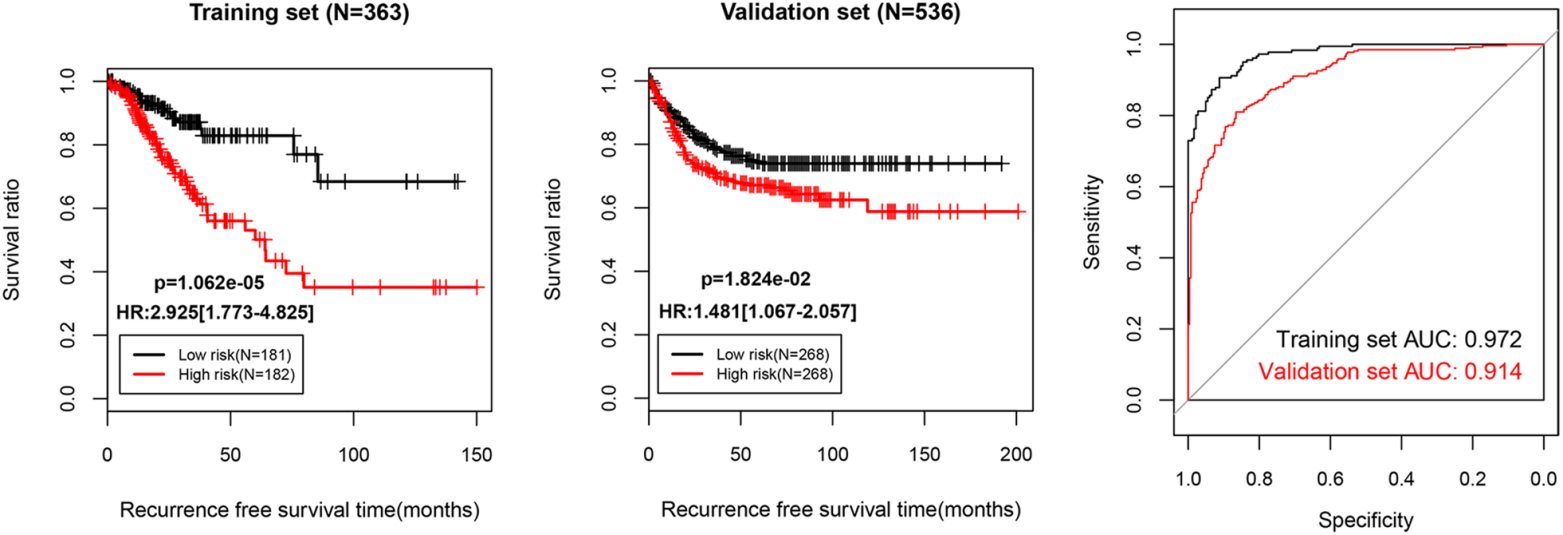
Validation of the 6 lncRNA-based prognostic signature. Based on the RS prediction model, prognostic related Kaplan–Meier curves were drawn in training set (left picture) and validation set (middle picture). The black and red curves represent low- and high-risk group, respectively (right picture). The ROC curve of RS prediction model; black and red curves represent the ROC curves of training dataset and validation dataset, respectively

For the training set, univariate Cox proportional hazards regression analyses revealed 42 DElncRNAs significantly correlated with overall survival among the 50 differentially expressed lncRNAs. Stepwise multivariable Cox proportional hazards regression analyses identified 7 independent risk DElncRNAs. These 7 DElncRNAs were entered into LASSO-based Cox-PH model. Under the parameter (lambda value is 0.8139 and the maximum value of CVL is -459.9254) (Fig. 2A), a total of 6 signature lncRNAs were found to be significantly and independently related to prognosis (Table 1). The gene prognostic coefficient was shown in Fig. 2B. We then constructed a prognostic signature based on the expression levels of these 6 signature lncRNAs and their coefficients derived from the multivariable Cox model. The RS of each patient in the training and validation datasets was calculated using the formula: RS = (1.0109537) × Exp_LINC00899_ + (0.6383124) × Exp_LINC01503_ + (-0.499666) × Exp_PRKAG2-AS1_ + (-1.977238) × Exp_RAD21-AS1_ + (2.0163898) × Exp_SRRM2-AS1_ + (-0.386197) × Exp_USP30-AS1_. According to the median risk score, the training and validation datasets were divided into a high-risk group and a low-risk group. Kaplan–Meier analysis (Fig. 3) revealed that the high-risk group had a significantly poorer RFS prognosis than that of the low-risk group in training dataset (log-rank *p* = 1.062e-05) and validation dataset (log-rank *p* = 1.824e-02). To evaluate the performance of the 6-lncRNA signature for predicting the prognosis of COAD patients, the ROC curve and the area under the ROC curve (AUC) were drawn. Meanwhile, the area under the AUC for the 6-lncRNA signature was 0.972 in training dataset and 0.914 in validation dataset, which indicated good performance.

**Table 1:** A 6-lncRNA signature significantly and independently correlated to prognosis

Symbol	coef	Pr ( >|z|)	Hazard Ratio	95%CI
LINC00899	1.0109537	0.041095	2.852	1.875–4.298
LINC01503	0.6383124	0.003025	2.150	1.245–3.712
PRKAG2-AS1	− 0.499666	0.000635	0.483	0.310–0.752
RAD21-AS1	− 1.977238	0.002405	0.040	0.018–0.186
SRRM2-AS1	2.0163898	0.01163	3.511	1.624–7.592
USP30-AS1	− 0.386197	0.04873	0.694	0.450–0.969

### Building nomogram based on prognostic clinical factors and the six-lncRNA signature

**Fig. 4:**
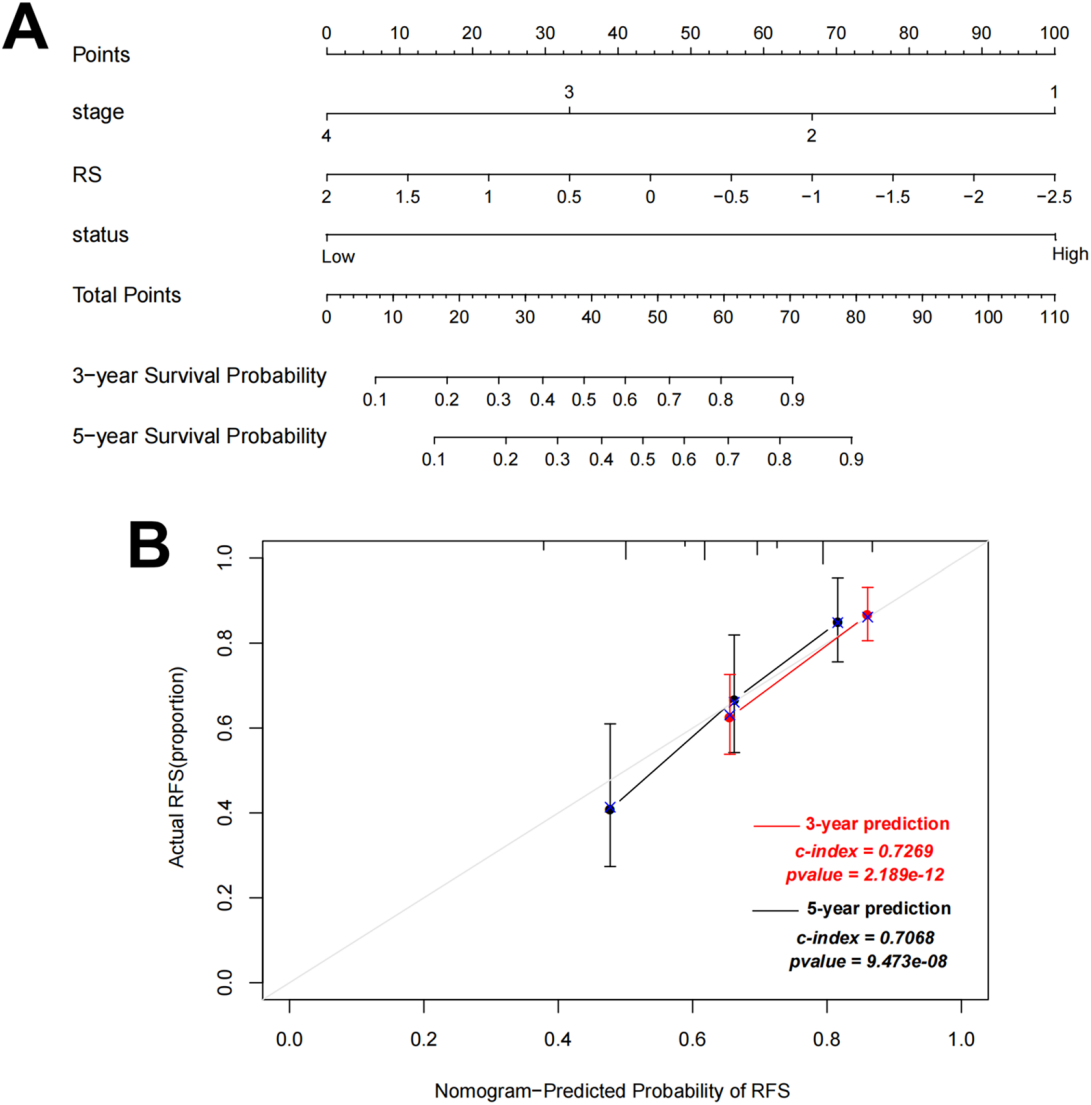
A nomogram incorporating risk score based on pathologic stage and RS model status for predicting survival of COAD patients. **A** The sum of points for each variable value is located on Total Point axis, and used to determine likelihood of 3-year and 5-year recurrence free survival of each individual patient. **B** Calibration plots of nomogram for predicting 1-year and 3-year recurrence free survival

Using univariate and multivariate regression analysis, we found that pathologic stage and RS model status were independent prognostic factors in the training dataset, as summarized in Table 2 and Additional file 2: Figure S1. Combining expression risk score based on the pathologic stage and RS model status, we constructed a nomogram to improve predictive accuracy (Fig. 4A). As shown in calibration plots (Fig. 4B), the predicted 3-year and 5-year RFS was consistent with the actual 3-year and 5-year RFS.

**Table 2:** Determination of prognostic clinical factors

Variables	Univariate analysis		Multivariate analysis	
	HR (95% CI)	P value	HR (95% CI)	P value
Age (years, mean ± SD)	0.989 (0.971–1.006)	1.937E−01	NA	NA
Gender (Male/Female)	1.630 (1.030–2.579)	3.521E−02*	1.567 (0.984–2.495)	5.872E−02
Pathologic M (M0/M1/−)	3.498 (2.015–6.072)	2.162E−06*	2.358 (0.870–6.391)	9.165E−02
Pathologic N (N0/N1/N2)	0.909 (0.537–1.540)	7.226E−01	NA	NA
Pathologic T (T1/T2/T3/T4)	2.310 (1.484–3.597)	3.167E−04*	1.552 (0.909–2.649)	1.070E−01
Pathologic stage (I/II/III/IV/−)	1.849 (1.423–2.402)	2.874E−06*	1.482 (1.105–1.986)	8.541E−03*
lncRNA RS model (High/Low)	2.925 (1.773–4.825)	1.062E−05*	2.438 (1.443–4.118)	8.610E−04*
Recurrence (Yes/No)	NA	NA	NA	NA
Recurrence free survival time (months, mean ± SD)	NA	NA	NA	NA

*Statistically significant;* RS* risk score,* HR* hazard ratio,* CI* confidence interval,* NA* not analyzed

### Construction of ceRNA regulatory network

**Fig. 5:**
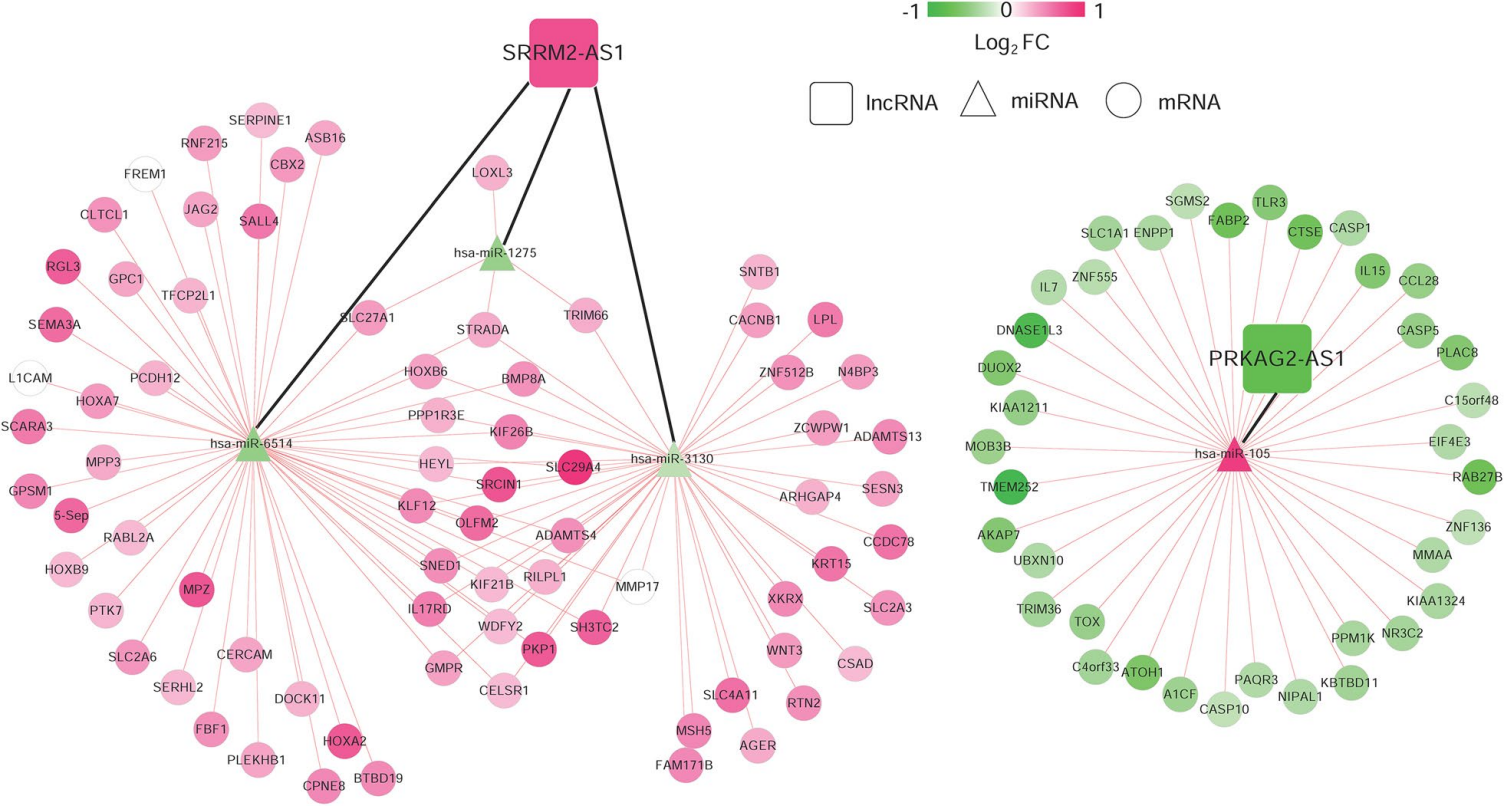
The DERs in ceRNA network. A global view of the ceRNA regulatory network in COAD. Rectangles, signature lncRNAs; triangles, DEmiRNAs; circles, DEmRNAs; A change in color from green to red indicates a change in significantly down-regulated to up-regulated expression of log_2_FC. The black and red lines represent signature lncRNA-prognostic miRNA connections and prognostic miRNA-mRNA regulatory connections, respectively

To explore the targeting relationship of the DERs, we focused on the interaction of 18 DEmiRNAs with 6-lncRNA signature and DEmRNAs. Firstly, we explored the regulatory loops with lncRNA-miRNA in the DIANA-LncBasev2 database and found that 2 of 6 specific DElncRNAs might target 4 of 18 specific DEmiRNAs. Subsequently, we screened 137 connection pairs between 4 DEmiRNAs and selected DEmRNAs predicted by (TargetScan, PicTar, RNA22, Pita and MIRANDA) (Additional file 3: Table S2). Finally, on account of the regulatory pairs of lncRNA signature-DEmiRNA and DEmiRNA-DEmRNA, we constructed the lncRNA-miRNA-mRNA ceRNA network using Cytoscape 3.6.1 software. In total, 2 lncRNAs, 4 miRNAs, and 113 mRNAs were included in the ceRNA regulatory network, containing 117 nodes and 137 edges (Fig. 5).

### Function enrichment analysis

According to the GSEA-based KEGG signaling pathway enrichment analysis in DERs from ceRNA network, we screened the KEGG pathways that were significantly correlated with two signature lncRNAs using NOM P value less than 0.05 as the threshold. As shown in enrichment plots, two KEGG signaling pathways (PPAR_SIGNALING_PATHWAY and CYTOKINE_CYTOKINE_RECEPTOR_INTERACTION) were screened to be significantly associated with PRKAG2-AS1 and SRRM2-AS1 (Additional file 4: Figure S2).

### SRRM2-AS1 was upregulated in COAD and high SRRM2-AS1 level predicted worse prognosis

**Fig. 6:**
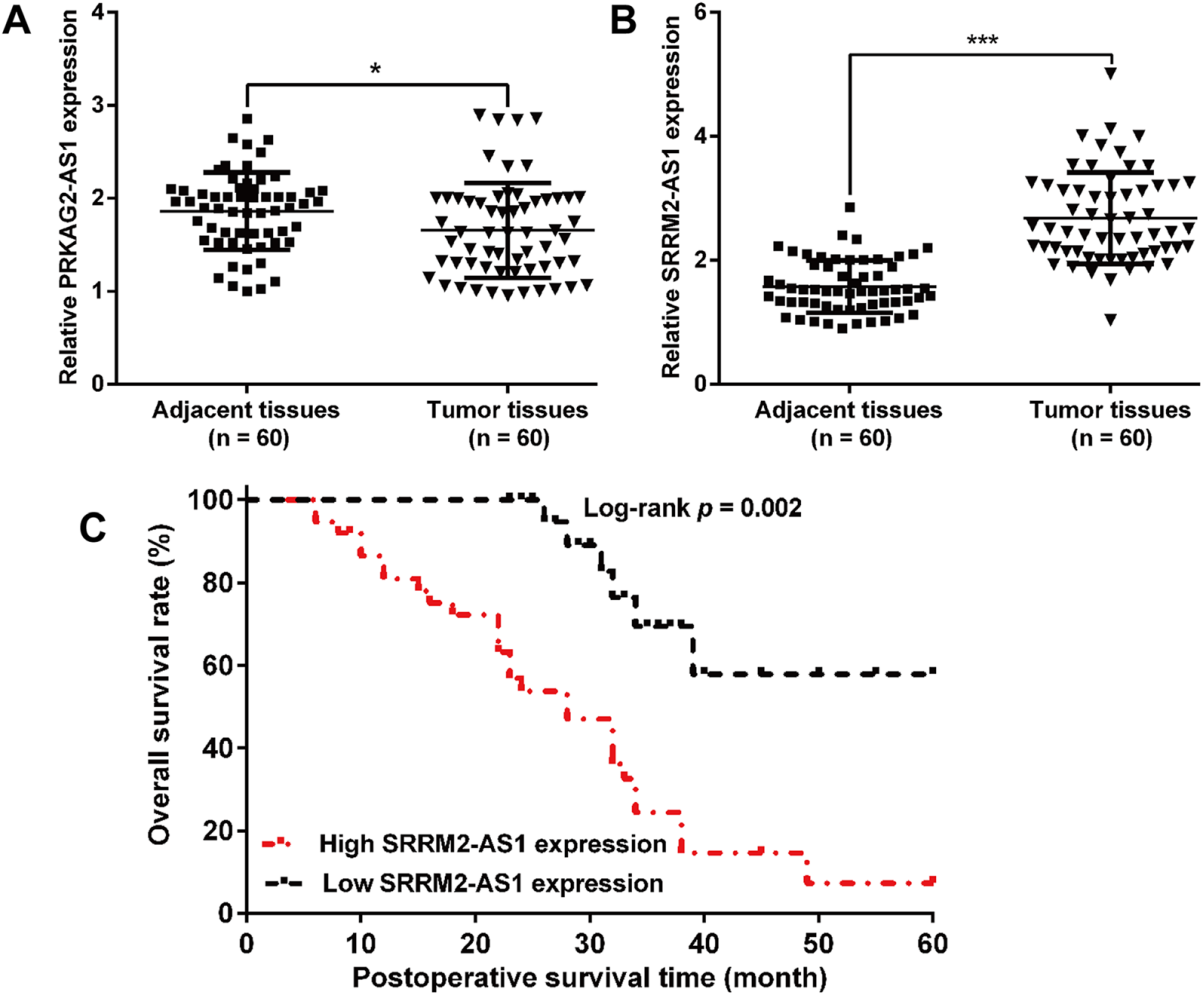
Relative SRRM2-AS1 expression and its prognostic value in COAD patients. The expression levels of PRKAG2-AS1 (**A**) and SRRM2-AS1 (**B**) were determined in 60 pairs of tumor and adjacent tissues derived from COAD patients using quantitative RT-PCR analysis. **C** Correlation between SRRM2-AS1 and overall survival of COAD patients was analyzed by Kaplan–Meier method (log-rank test: *p* = 0.002)

We initially validated the expression of PRKAG2-AS1 and SRRM2-AS1 in 60 pairs of tumor tissues and adjacent tissues derived from COAD patients. The results from quantitative RT-PCR showed that PRKAG2-AS1 (Fig. 6A) was downregulated, while SRRM2-AS1 (Fig. 6B) was upregulated in tumor tissues compared with adjacent tissues, which represented the same results by analyzing the TCGA-COAD dataset. Considering the relatively higher fold change in SRRM2-A1 expression, SRRM2-AS1 was selected for further analysis. By dividing all patients into high and low expression group with the median value of SRRM2-AS1 as a cutoff value, we found high level of SRRM2-AS1 was significantly associated with tumor size, lymph node metastasis and TNM stage (Table 3). Kaplan–Meier analysis revealed that patients with low-level SRRM2-AS1 had better overall survival than those with high-level SRRM2-AS1 (Fig. 6C, log-rank test; *p* = 0.002). More importantly, high SRRM2-AS1 expression was identified as an independent unfavorable prognostic factor in COAD patients through univariate and multivariate Cox regression analysis (Table 4). These results indicated that SRRM2-AS1 may be involved in the malignant progression of COAD.

**Table 3:** Association between SRRM2-AS1 expression and clinicopathological characteristics of COAD patients

Characteristics	Cases (n = 60)	SRRM2-AS1 expression		*P* value
		Low (n = 22)	High (n = 38)	(chi-square test)
Age				0.310
< 55	26	10	16	
≥ 55	34	12	22	
Gender				0.746
Male	32	9	23	
Female	28	13	15	
Tumor size (cm)				0.031*
< 5	38	16	22	
≥ 5	22	6	16	
Lymph node metastasis				0.023*
Negative	35	15	20	
Positive	25	7	18	
TNM stage				0.009*
I–II	33	15	18	
III–IV	27	7	20	
Differentiation				0.295
Well/moderately	42	15	27	
Poorly	18	7	11	

*TNM* tumor‑node‑metastasis classification system

*Indicates *p*-value less than 0.05 that recognized as statistical significance

**Table 4:** Cox regression analysis of prognostic predictors affecting overall survival in COAD patients

Characteristics	Univariate analysis		Multivariate analysis	
	HR (95% CI)	P value	HR (95% CI)	P value
Age	1.102 (0.835–1.998)	0.375	NA	NA
Gender	0.895 (0.486–1.278)	0.812	NA	NA
Tumor size (cm)	3.214 (2.278–4.325)	0.023*	2.845 (1.696–3.142)	0.018*
Lymph node metastasis	2.013 (1.843–3.204)	0.028*	1.734 (0.996–2.512)	0.042*
TNM stage	1.421 (1.046–2.417)	0.013*	1.989 (1.296–2.751)	0.051
Differentiation	1.204 (0.712–2.048)	0.475	NA	NA
SRRM2-AS1 expression	0.998 (0.546–1.312)	0.013*	0.853 (0.496–1.143)	0.024*

*HR* hazard ratio, *CI* confidence interval, *TNM* tumor‑node‑metastasis, *NA* not analyzed

*Indicates *p*-value less than 0.05 that recognized as statistical significance

### Knockdown of SRRM2-AS1 suppressed the COAD cell proliferation, migration and invasion

**Fig. 7:**
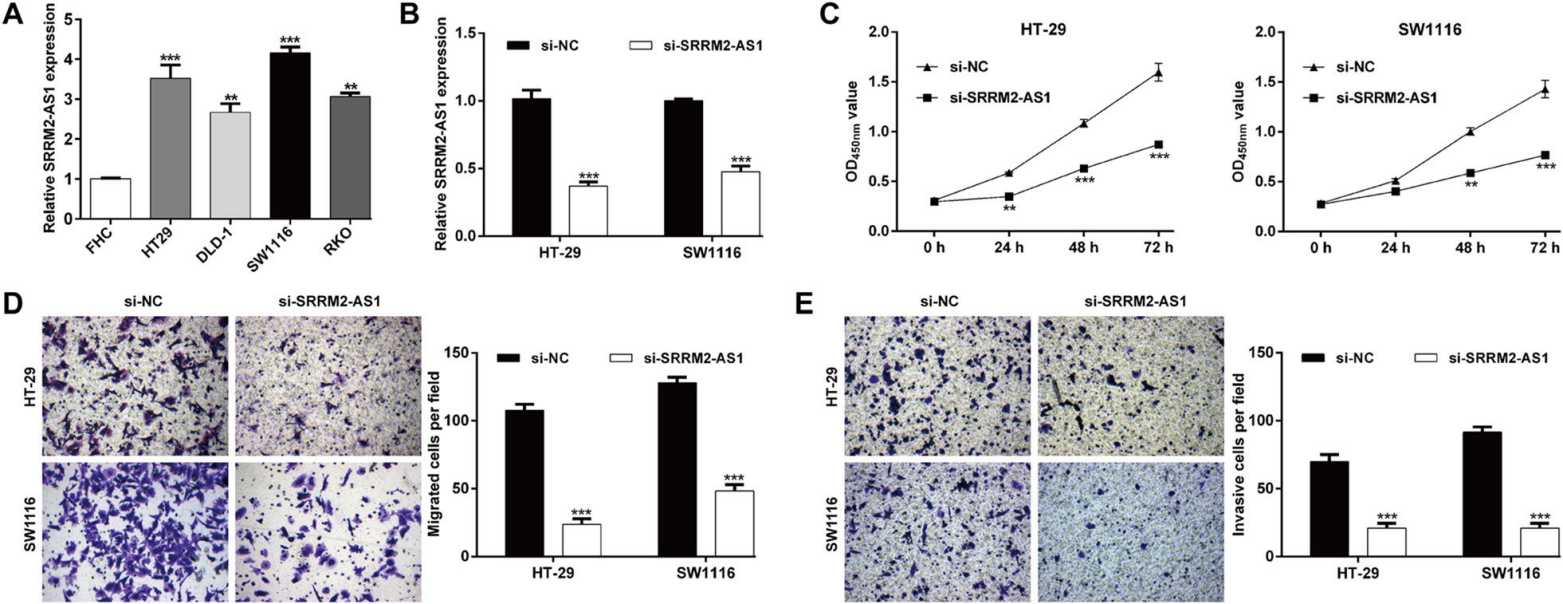
Knockdown of SRRM2-AS1 suppressed the COAD cell proliferation, migration and invasion. **A** The SRRM2-AS1 expression profile in primary COAD cell lines (HT-29, DLD-1, SW1116 and RKO) and normal human colon epithelial cell line (FHC). **B** Transfection efficiency of SRRM2-AS1 siRNA was determined by PCR. **C** The proliferative ability of HT-29 and SW1116 cells was determined by CCK-8 assay. Transwell assay was performed to analyze cell migration (**D**) and invasion (**E**) in transfected HT-29 and SW1116 cells. Data are presented as means ± SD. **p < 0.01, ***p < 0.001, compared with FHC or si-NC

To further explore the biological function of SRRM2-AS1 on COAD in vitro, we first determined the expression of SRRM2-AS1 in several COAD cell lines. As shown in Fig. 7A, primary COAD cell lines (HT-29, DLD-1, SW1116 and RKO) expressed higher SRRM2-AS1 levels compared with normal human colon epithelial cell line (FHC). Next, si-SRRM2-AS1 was transfected into HT-29 and SW1116 cells, which significantly suppressed the expression of SRRM2-AS1 (Fig. 7B). Knockdown of SRRM2-AS1 significantly inhibited the proliferation ability of HT-29 and SW1116 cells (Fig. 7C). In addition, the migratory (Fig. 7D) and invasive (Fig. 7E) capacities of HT-29 and SW1116 cells were also repressed after si-SRRM2-AS1 transfection, in comparison with si-NC transfection. These results indicated that SRRM2-AS1 can promote the growth and metastasis of COAD in vitro.

### Exploration on the molecular mechanism underlying SRRM2-AS1 knockdown on COAD cells

**Fig. 8:**
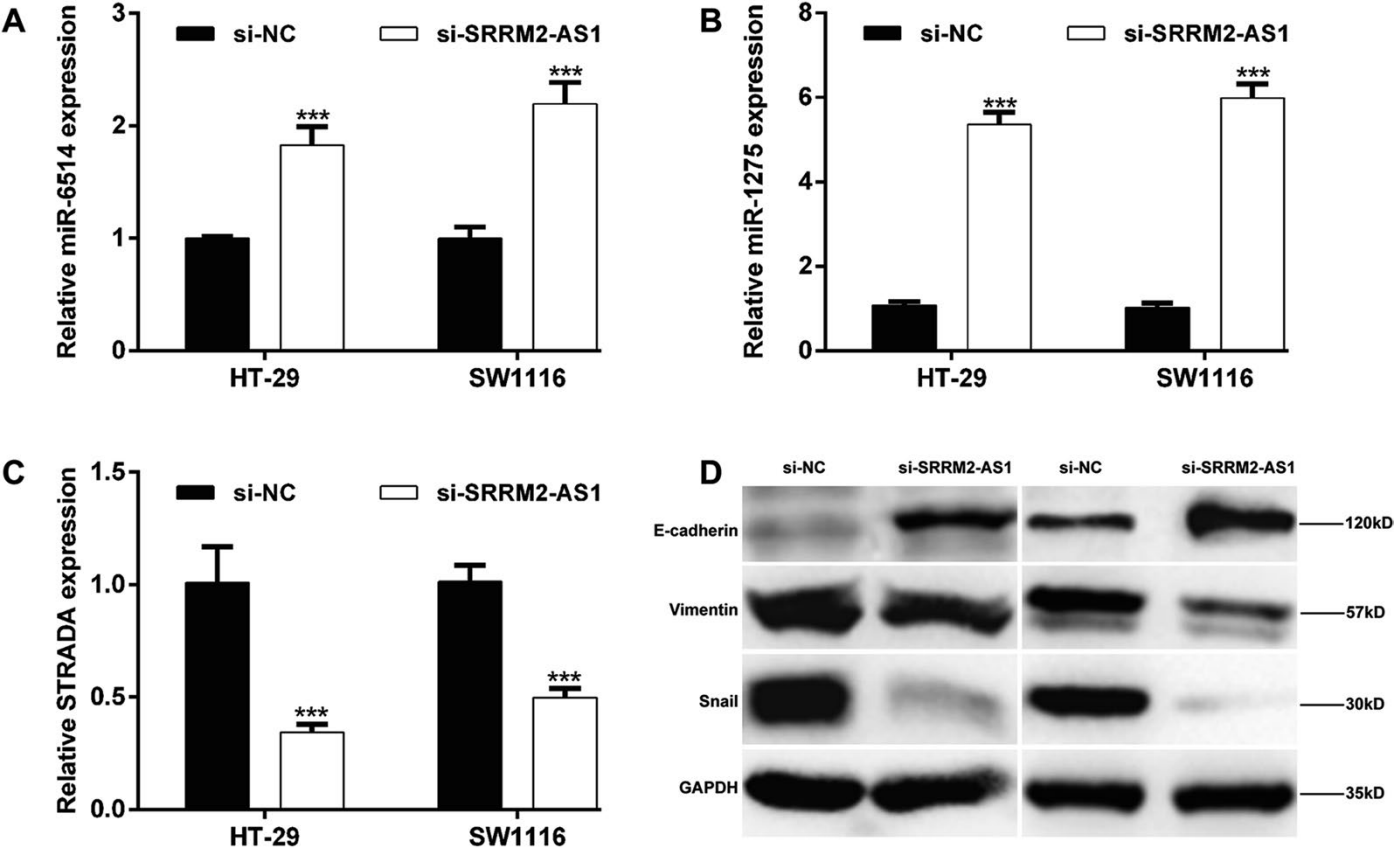
Exploration on the molecular mechanism underlying SRRM2-AS1 knockdown on COAD cells. HT-29 and SW1116 cells were transfected with si-SRRM2-AS1. Quantitative RT-PCR analysis was performed to determine the expression levels of **A** miR-6514, **B** miR-1275 and **C** STRADA in transfected HT-29 and SW1116 cells. Data are presented as means ± SD. ***p < 0.001, compared with si-NC; **D** The protein expression levels of E-cadherin, Vimentin and Snail were detected by western blot analysis

According to the predicted ceRNA regulatory network, we selected several miRNAs and mRNA targets of SRRM2-AS1 to analyze their expression levels under SRRM2-AS1 knockdown in COAD cells. The results showed that knockdown of SRRM2-AS1 significantly upregulated the expression levels of miR-6514 (Fig. 8A) and miR-1275 (Fig. 8B), while downregulated STRADA mRNA level (Fig. 8C) in both HT-29 and SW1116 cells. In addition, we measured the protein levels of EMT markers. As shown in Fig. 8D, SRRM2-AS1 knockdown increased E-cadherin expression, while decreased the protein expression of Vimentin and Snail in HT-29 and SW1116 cells.

## Discussion

With the development of high-throughput sequencing technology, increasing amounts of sequencing data have been used for studies of cancer diagnosis, therapy, and prognosis [34]. Here, we downloaded RNA-seq data and relevant clinical data related to COAD from TCGA database and obtained 363 COAD samples with recurrence information. A total of 411 DERs, including 18 DEmiRNAs (9 up-regulated and 9 down-regulated), 338 DEmRNAs (225 up-regulated and 113 down-regulated) and 55 DElncRNAs (43 up-regulated and 12 down-regulated) were identified in recurrence samples compared with non-recurrence samples. Subsequently, the correlations between DElncRNAs and RFS prognosis were identified to establish a RS model for predicting COAD recurrent prognosis. Accordingly, a 6-lncRNA signature risk prediction model (LINC00899, LINC01503, PRKAG2-AS1, RAD21-AS1, SRRM2-AS1 and USP30-AS1) was produced. Patients were sub-divided into high- and low-risk groups based on the median risk score. The AUC values for the time-dependent ROC curve in the training and validation dataset were 0.972 and 0.914, respectively, indicating outstanding performance in survival prediction. According to the identified pathologic stage and RS model status as the independent RFS prognostic factors, we built the 3-year and 5-year nomogram survival model and validated its consistence with actual 3-year and 5-year RFS.

By searching published articles on these six-lncRNA signature in tumor development, we found that LINC00899 is elevated in the serum and bone marrow of acute myeloid leukemia (AML) patients and high serum LINC00899 expression was an independent prognostic marker of poor outcome [35]. Dong et al. [36] further demonstrated that LINC00899 promoted cell proliferation and inhibited apoptosis in AML cells. LINC01503 expression level was significantly up-regulated, correlated with poor prognosis and conferred oncogenic functions in glioma [37], hepatocellular carcinoma [38], gastric cancer [39] and especially colorectal cancer [40]. Silencing of PRKAG2-AS1 alleviated castration-resistant prostate cancer (CRPC) tumor growth, showing repression of androgen receptor (AR) and AR variant expression [41]. In addition, RAD21-AS1 [42], SRRM2-AS1 [43] and USP30-AS1 [44, 45] have been reported to be survival prognosis in various cancers. These suggested that identified 6-lncRNA signature might be involved in the recurrence prognosis of COAD.

On the basis of the identified lncRNA signature, DEmiRNAs and DEmRNAs, we constructed lncRNA-miRNA-mRNA ceRNA network of COAD, of which 2 lncRNAs, 4 miRNAs, and 113 mRNAs were included. Notably, SRRM2-AS1 binds to the target miR-1275, miR-6514 and miR-3130, while PRKAG2-AS1 binds to the target miR-105 (Additional file 4: Fig. S2). These data indicated that PRKAG2-AS1 and SRRM2-AS1 might play roles in the recurrence prognosis of COAD by regulating their corresponding target miRNAs. Except for miR-6514 and miR-3130, accumulating evidence has revealed the functional roles of miR-1275 [46, 47] and miR-105 [48, 49] in the development of tumorigenesis. The function enrichment analysis identified the PPAR_SIGNALING_PATHWAY and CYTOKINE_CYTOKINE_RECEPTOR_INTERACTION significantly associated with PRKAG2-AS1 and SRRM2-AS1, which might be the recurrence prognosis of COAD. Consistent with our findings, Jansson et al. [50] reported that peroxisome proliferator activated receptor gamma (PPAR gamma) protein levels are elevated, possibly through interaction with beta-catenin and T cell transcription factor-4 in colon cancer cell lines. CYTOKINE_CYTOKINE_RECEPTOR_INTERACTION pathway has been reported to promote tumor progression in models of colorectal cancer, which predicts unfavorable outcomes in colon cancer patients [51, 52].

Moreover, we harvested 60 pairs of COAD tissues and adjacent tissues. After a series of clinical analysis, we confirmed that high SRRM2-AS1 expression was identified as an independent unfavorable prognostic factor in COAD patients. Functional experiments further manifested that knockdown of SRRM2-AS1 suppressed the cell proliferation, migration and invasion in two COAD cell lines (HT-29 and SW1116). Moreover, SRRM2-AS1 knockdown suppressed the epithelial-to-mesenchymal transition process, as reflected by increased E-cadherin expression and decreased Vimentin and Snail expression levels. Similar to our study design, HOTAIR depletion could reduce cellular motility, invasiveness and EMT in human tumor cells [53]. Furthermore, silencing of SRRM2-AS exerts suppressive effects on angiogenesis in nasopharyngeal carcinoma [54]. As our best knowledge, SRRM2-AS1 has not been explored on its biological function in tumor cells, except for a recent study by Yang et al. [43] who also identified SRRM2-AS1 as an independent prognosis-associated lncRNA for predicting the recurrence of COAD patients. Anyway, our experiment validation is far from adequate, including lacking of deeper functional analysis on these miRNAs, their interactions and in vivo animal experiments and deeper molecular mechanism exploration.

## Conclusion

In summary, we identified differentially expressed gene associated with the recurrent prognosis of COAD patients and constructed a 6 lncRNA prognostic model to predict prognosis of patients. The prognostic model presented a good performance in 3- and 5-year RFS prediction. Importantly, we have successfully constructed a lncRNA-associated ceRNA network, which provides novel lncRNAs as candidate potential therapeutic targets for associated with independent recurrent prognosis in COAD.

## Supplementary Information

**Additional file 1: Table S1.** All identified DERs between recurrence samples and non-recurrence samples.

**Additional file 2: Figure S1.** Kaplan-Meier curves for recurrence free survival of patients in the training set classified by pathologic stage. All patients in the training dataset are divided by stage into two subgroups, respectively. The patients in early stage have significantly longer recurrence free survival time than the patients in advanced stage.

**Additional file 3: Table S2. **Screening of connection pairs between 4 DEmiRNAs and selected DEmRNAs predicted by (TargetScan, PicTar, RNA22, Pita and MIRANDA).

**Additional file 4: Figure S2. **Partial display of the GSEA analysis results. Enrichment plot: PPAR_SIGNALING_PATHWAY and CYTOKINE_CYTOKINE_RECEPTOR_INTERACTION associated with PRKAG2-AS1 and SRRM2-AS1.

## Data Availability

The datasets used and/or analyzed during the current study are available from the corresponding author on reasonable request.

## Abbreviations

lncRNAs: Long non-coding RNAs
ceRNA: Competing endogenous RNA
COAD: Colon adenocarcinoma
DERs: Differentially expressed RNAs
RS: Risk-score
CVL: Cross-validation likelihood
AML: Acute myeloid leukemia
CRPC: Castration-resistant prostate cancer

## References

[1] Siegel RL, Miller KD, Jemal A (2019). Cancer statistics, 2019. CA Cancer J Clin.

[2] Maley CC, Aktipis A, Graham TA, Sottoriva A, Boddy AM, Janiszewska M (2017). Classifying the evolutionary and ecological features of neoplasms. Nat Rev Cancer.

[3] Obaro AE, Burling DN, Plumb AA (2018). Colon cancer screening with CT colonography: logistics, cost-effectiveness, efficiency and progress. Br J Radiol.

[4] Mutch MG (2007). Molecular profiling and risk stratification of adenocarcinoma of the colon. J Surg Oncol.

[5] Renehan AG, O'Dwyer ST, Haboubi NJ, Potten CS (2002). Early cellular events in colorectal carcinogenesis. Colorectal Dis.

[6] Mejri N, Dridi M, El Benna H, Labidi S, Daoud N, Boussen H (2018). Tumor location impact in stage II and III colon cancer: epidemiological and outcome evaluation. J Gastrointest Oncol.

[7] Favoriti P, Carbone G, Greco M, Pirozzi F, Pirozzi RE, Corcione F (2016). Worldwide burden of colorectal cancer: a review. Updates Surg.

[8] Mody K, Bekaii-Saab T (2018). Clinical trials and progress in metastatic colon cancer. Surg Oncol Clin N Am.

[9] Fatica A, Bozzoni I (2014). Long non-coding RNAs: new players in cell differentiation and development. Nat Rev Genet.

[10] Castro-Oropeza R, Melendez-Zajgla J, Maldonado V, Vazquez-Santillan K (2018). The emerging role of lncRNAs in the regulation of cancer stem cells. Cell Oncol (Dordr).

[11] Yan X, Hu Z, Feng Y, Hu X, Yuan J, Zhao SD (2015). Comprehensive genomic characterization of long non-coding RNAs across human cancers. Cancer Cell.

[12] Jing N, Huang T, Guo H, Yang J, Li M, Chen Z (2018). LncRNA CASC15 promotes colon cancer cell proliferation and metastasis by regulating the miR4310/LGR5/Wnt/betacatenin signaling pathway. Mol Med Rep.

[13] Wang L, Wei Z, Wu K, Dai W, Zhang C, Peng J (2018). Long noncoding RNA B3GALT5-AS1 suppresses colon cancer liver metastasis via repressing microRNA-203. Aging (Albany NY).

[14] Fu X, Duanmu J, Li T, Jiang Q (2020). A 7-lncRNA signature associated with the prognosis of colon adenocarcinoma. PeerJ.

[15] Lin Y, Pan X, Chen Z, Lin S, Chen S (2020). Identification of an immune-related nine-lncRNA signature predictive of overall survival in colon cancer. Front Genet.

[16] Zhou W, Zhang S, Li HB, Cai Z, Tang S, Chen LX (2020). Development of prognostic indicator based on autophagy-related lncRNA analysis in colon adenocarcinoma. Biomed Res Int.

[17] Salmena L, Poliseno L, Tay Y, Kats L, Pandolfi PP. A ceRNA hypothesis: the Rosetta Stone of a hidden RNA language? Cell. 2011;146(3):353–358. 10.1016/j.cell.2011.07.014 PMC3235919 21802130

[18] Wu Q, Meng WY, Jie Y, Zhao H (2018). LncRNA MALAT1 induces colon cancer development by regulating miR-129-5p/HMGB1 axis. J Cell Physiol.

[19] Liu J, Zhan Y, Wang J, Wang J, Guo J, Kong D (2020). lncRNA-SNHG17 promotes colon adenocarcinoma progression and serves as a sponge for miR-375 to regulate CBX3 expression. Am J Transl Res.

[20] Wright MW (2014). A short guide to long non-coding RNA gene nomenclature. Hum Genomics.

[21] Ritchie ME, Phipson B, Wu D, Hu Y, Law CW, Shi W (2015). limma powers differential expression analyses for RNA-sequencing and microarray studies. Nucleic Acids Res.

[22] Eisen MB, Spellman PT, Brown PO, Botstein D (1998). Cluster analysis and display of genome-wide expression patterns. Proc Natl Acad Sci U S A.

[23] Wang L, Cao C, Ma Q, Zeng Q, Wang H, Cheng Z (2014). RNA-seq analyses of multiple meristems of soybean: novel and alternative transcripts, evolutionary and functional implications. BMC Plant Biol.

[24] Wang P, Wang Y, Hang B, Zou X, Mao JH (2016). A novel gene expression-based prognostic scoring system to predict survival in gastric cancer. Oncotarget.

[25] Tibshirani R (1997). The lasso method for variable selection in the Cox model. Stat Med.

[26] Goeman JJ (2010). L1 penalized estimation in the Cox proportional hazards model. Biom J.

[27] Anderson WI, Schlafer DH, Vesely KR (1989). Thyroid follicular carcinoma with pulmonary metastases in a beaver (*Castor canadensis*). J Wildl Dis.

[28] Eng KH, Schiller E, Morrell K (2015). On representing the prognostic value of continuous gene expression biomarkers with the restricted mean survival curve. Oncotarget.

[29] Paraskevopoulou MD, Vlachos IS, Karagkouni D, Georgakilas G, Kanellos I, Vergoulis T (2016). DIANA-LncBase v2: indexing microRNA targets on non-coding transcripts. Nucleic Acids Res.

[30] Li JH, Liu S, Zhou H, Qu LH, Yang JH (2014). starBase v2.0: decoding miRNA-ceRNA, miRNA-ncRNA and protein-RNA interaction networks from large-scale CLIP-Seq data. Nucleic Acids Res.

[31] Shannon P, Markiel A, Ozier O, Baliga NS, Wang JT, Ramage D (2003). Cytoscape: a software environment for integrated models of biomolecular interaction networks. Genome Res.

[32] Subramanian A, Tamayo P, Mootha VK, Mukherjee S, Ebert BL, Gillette MA (2005). Gene set enrichment analysis: a knowledge-based approach for interpreting genome-wide expression profiles. Proc Natl Acad Sci USA.

[33] Zheng L, Pu J, Qi T, Qi M, Li D, Xiang X (2013). miRNA-145 targets v-ets erythroblastosis virus E26 oncogene homolog 1 to suppress the invasion, metastasis, and angiogenesis of gastric cancer cells. Mol Cancer Res.

[34] Zou AE, Ku J, Honda TK, Yu V, Kuo SZ, Zheng H (2015). Transcriptome sequencing uncovers novel long noncoding and small nucleolar RNAs dysregulated in head and neck squamous cell carcinoma. RNA.

[35] Wang Y, Li Y, Song HQ, Sun GW (2018). Long non-coding RNA LINC00899 as a novel serum biomarker for diagnosis and prognosis prediction of acute myeloid leukemia. Eur Rev Med Pharmacol Sci.

[36] Dong X, Xu X, Guan Y (2020). LncRNA LINC00899 promotes progression of acute myeloid leukaemia by modulating miR-744-3p/YY1 signalling. Cell Biochem Funct.

[37] Wang H, Sheng ZG, Dai LZ (2019). Long non-coding RNA LINC01503 predicts worse prognosis in glioma and promotes tumorigenesis and progression through activation of Wnt/β-catenin signaling. Eur Rev Med Pharmacol Sci.

[38] Wang MR, Fang D, Di MP, Guan JL, Wang G, Liu L (2020). Long non-coding RNA LINC01503 promotes the progression of hepatocellular carcinoma via activating MAPK/ERK pathway. Int J Med Sci.

[39] Ding J, Shi F, Xie G, Zhu Y (2020). Long non-coding RNA LINC01503 promotes gastric cancer cell proliferation and invasion by regulating Wnt signaling. Dig Dis Sci.

[40] Lu SR, Li Q, Lu JL, Liu C, Xu X, Li JZ (2018). Long non-coding RNA LINC01503 promotes colorectal cancer cell proliferation and invasion by regulating miR-4492/FOXK1 signaling. Exp Ther Med.

[41] Takayama KI, Fujimura T, Suzuki Y, Inoue S (2020). Identification of long non-coding RNAs in advanced prostate cancer associated with androgen receptor splicing factors. Commun Biol.

[42] Evans MF, Vacek PM, Sprague BL, Stein GS, Stein JL, Weaver DL (2020). Microarray and RNA in situ hybridization assay for recurrence risk markers of breast carcinoma and ductal carcinoma in situ: evidence supporting the use of diverse pathways panels. J Cell Biochem.

[43] Yang H, Lin HC, Liu H, Gan D, Jin W, Cui C (2020). A 6 lncRNA-based risk score system for predicting the recurrence of colon adenocarcinoma patients. Front Oncol.

[44] Chen P, Gao Y, Ouyang S, Wei L, Zhou M, You H (2020). A prognostic model based on immune-related long non-coding RNAs for patients with cervical cancer. Front Pharmacol.

[45] Tong H, Li T, Gao S, Yin H, Cao H, He W (2021). An epithelial-mesenchymal transition-related long noncoding RNA signature correlates with the prognosis and progression in patients with bladder cancer. Biosci Rep.

[46] Liu MD, Wu H, Wang S, Pang P, Jin S, Sun CF (2018). MiR-1275 promotes cell migration, invasion and proliferation in squamous cell carcinoma of head and neck via up-regulating IGF-1R and CCR7. Gene.

[47] He J, Yu L, Wang CM, Zhou XF (2018). MiR-1275 promotes non-small cell lung cancer cell proliferation and metastasis by regulating LZTS3 expression. Eur Rev Med Pharmacol Sci.

[48] Shang JC, Yu GZ, Ji ZW, Wang XQ, Xia L (2019). MiR-105 inhibits gastric cancer cells metastasis, epithelial-mesenchymal transition by targeting SOX9. Eur Rev Med Pharmacol Sci.

[49] Zhang HY, Ma JH (2020). miR-105 promotes the progression and predicts the prognosis for oral squamous cell carcinoma (OSCC). Cancer Manag Res.

[50] Jansson EA, Are A, Greicius G, Kuo IC, Kelly D, Arulampalam V (2005). The Wnt/beta-catenin signaling pathway targets PPARgamma activity in colon cancer cells. Proc Natl Acad Sci U S A.

[51] McCuaig S, Barras D, Mann EH, Friedrich M, Bullers SJ, Janney A (2020). The interleukin 22 pathway interacts with mutant KRAS to promote poor prognosis in colon cancer. Clin Cancer Res.

[52] Jung B, Staudacher JJ, Beauchamp D (2017). Transforming growth factor β superfamily signaling in development of colorectal cancer. Gastroenterology.

[53] Battistelli C, Garbo S, Riccioni V, Montaldo C, Santangelo L, Vandelli A (2021). Design and functional validation of a mutant variant of the LncRNA HOTAIR to counteract snail function in epithelial-to-mesenchymal transition. Cancer Res.

[54] Chen S, Lv L, Zhan Z, Wang X, You Z, Luo X (2020). Silencing of long noncoding RNA SRRM2-AS exerts suppressive effects on angiogenesis in nasopharyngeal carcinoma via activating MYLK-mediated cGMP-PKG signaling pathway. J Cell Physiol.

