# Supplementary figures and images for: Integrative analysis of ceRNA network reveals functional lncRNAs associated with independent recurrent prognosis in colon adenocarcinoma
Source: Cancer Cell Int. 2021 Jul 5;21:352. doi: 10.1186/s12935-021-02069-6 (PMC8259330; doi:10.1186/s12935-021-02069-6)

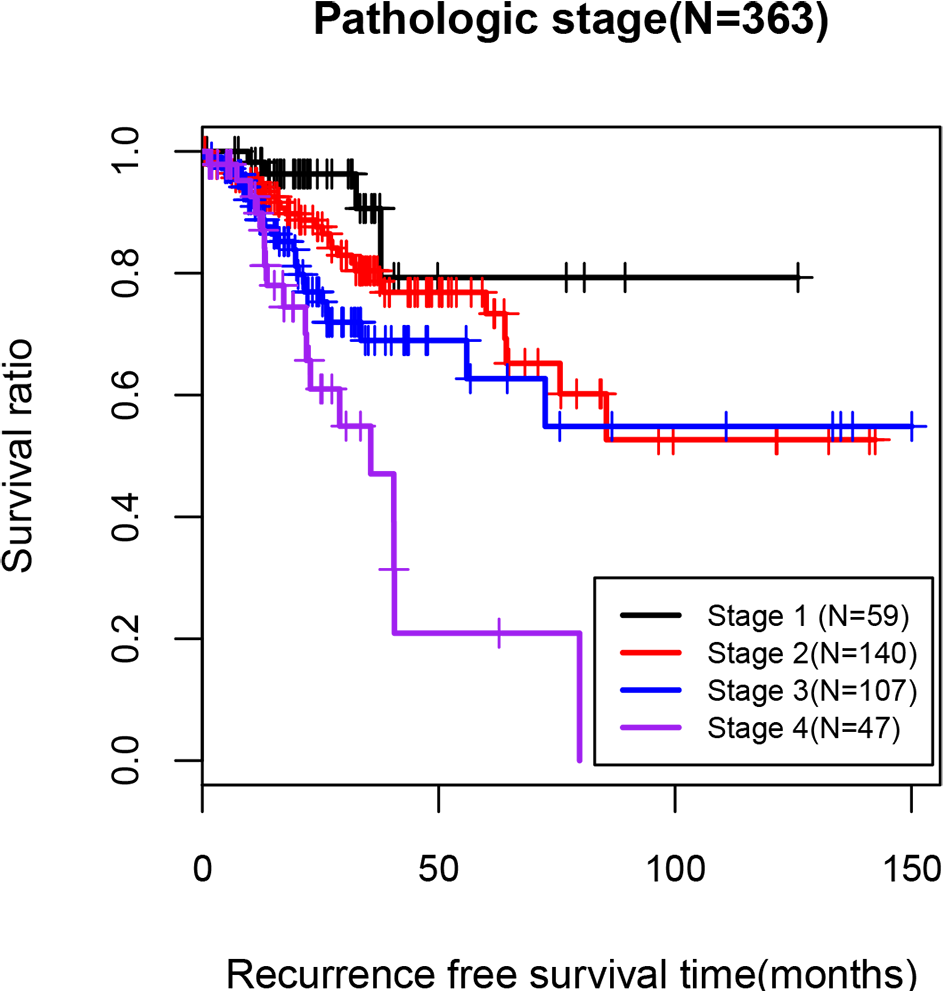

Supplement: Supplementary file 2 — Additional file 2: Figure S1. Kaplan-Meier curves for recurrence free survival of patients in the training set classified by pathologic stage. All patients in the training dataset are divided by stage into two subgroups, respectively. The patients in early stage have significantly longer recurrence free survival time than the patients in advanced stage. [file 12935_2021_2069_MOESM2_ESM.tif]

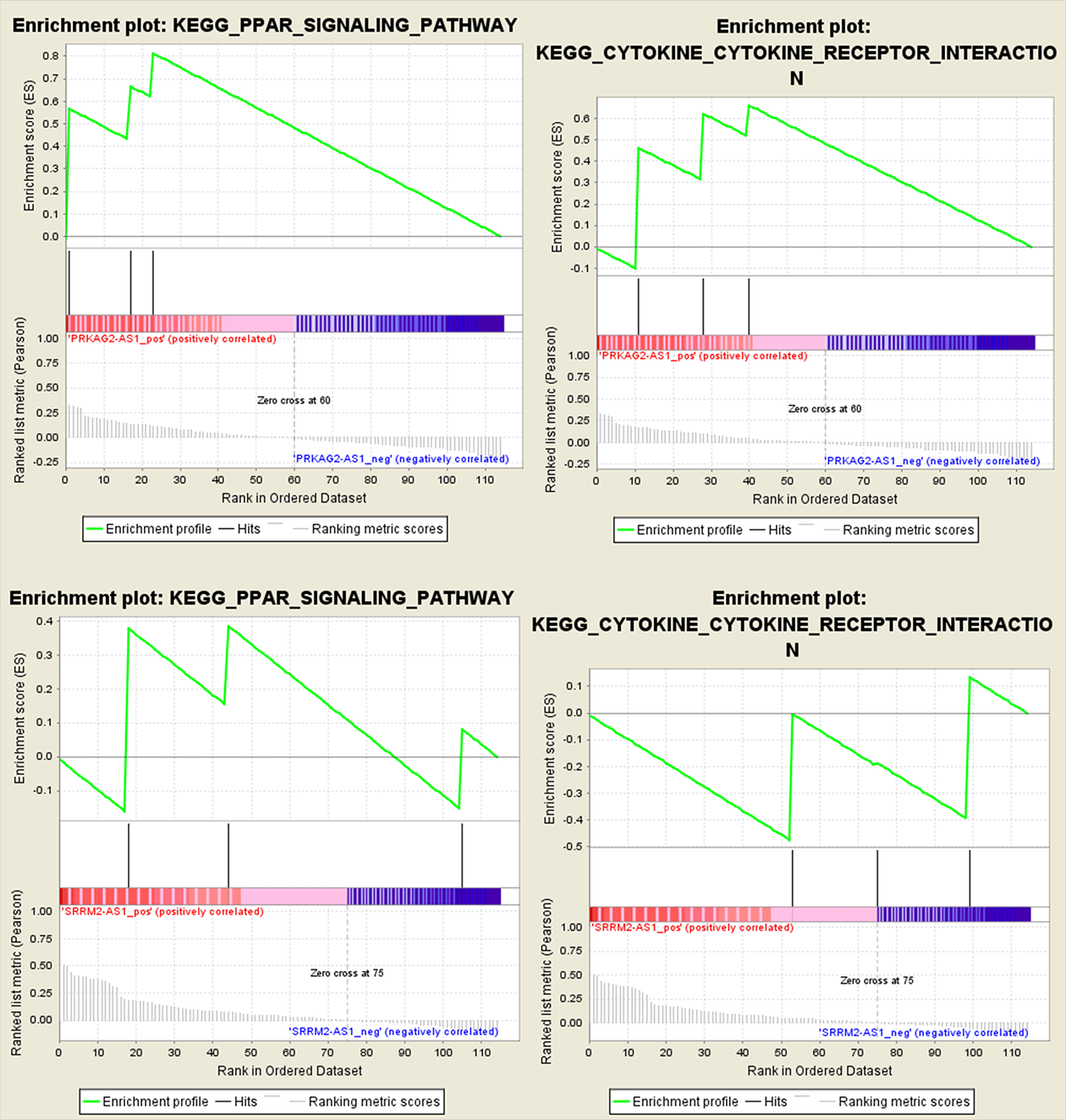

Supplement: Supplementary file 4 — Additional file 4: Figure S2. Partial display of the GSEA analysis results. Enrichment plot: PPAR_SIGNALING_PATHWAY and CYTOKINE_CYTOKINE_RECEPTOR_INTERACTION associated with PRKAG2-AS1 and SRRM2-AS1. [file 12935_2021_2069_MOESM4_ESM.tif]
